# Benchmarking the AlignRT surface deformation module for the early detection and quantification of oedema in breast cancer radiotherapy

**DOI:** 10.1016/j.tipsro.2021.12.002

**Published:** 2022-01-15

**Authors:** Veronica Sorgato, Khaoula Ghazouani, Yann Queffelec, Frederic Julia, Sophie Clement, Daniele Fric, Jad Farah

**Affiliations:** a Department of Radiotherapy, Daniel Hollard Cancer Institute, Grenoble, France; b School of Medical Physics, Faculty of Medicine, Grenoble Alpes University, France; c Vision RT Ltd., Dove House, Arcadia Ave, Finchley, London N3 2JU, United Kingdom

**Keywords:** Breast cancer, Oedema detection, SGRT, Surface deformation

## Abstract

**Purpose:**

To determine the accuracy of AlignRT surface deformation module in detecting and quantifying oedema in breast cancer radiotherapy.

**Materials and Methods:**

A female torso phantom and water-equivalent boluses of different thicknesses (0.5–1.5 cm) were used. The variation of surface displacement and the percentage of surface within tolerance, as a function of bolus thickness and Region of Interest (ROI) size, were investigated. Additionally, a dynamic phantom was used to study the impact of patient breathing on the swelling estimation. Lastly, a flowchart was derived to alert physicians in the case of breast swelling.

**Results:**

Average displacement value proved to be inversely correlated with ROI size (R^2^ > 0.9). As such, for a ROI smaller than the bolus size (2.5x2.5 cm^2^), the average displacement (1.05 cm) provides an accurate estimate of the oedema thickness (within 5%). In opposition, with a clinical ROI, the 1 cm-thick bolus was largely underestimated with an average displacement value of 0.28 cm only. To limit the impact of patient breathing on surface deformation, dynamic surface captures and the use of the corrected patient position should be privileged. Using AlignRT, a clinical workflow for breast swelling follow-up was developed to help in the decision for repeat simulation and dosimetry.

**Conclusion:**

The surface deformation module provides an accurate, simple, and radiation-free approach to detect and quantify breast oedema during the course of radiotherapy.

## Introduction

Adjuvant radiotherapy is a standard of care for breast cancer that improves locoregional control, and overall survival [1], [2], [3]. However, acute skin toxicity is very common and ranges from mild erythema to skin desquamation [4], [5]. Additionally, anatomical changes such as breast oedema during the course of radiotherapy is significant for conventional and hypo-fractionated treatments [6]. These require careful monitoring as they can affect the accuracy of dose delivery. Indeed, weight loss/gain, changes in breast size and shape, oedema, seroma, etc. [7], [8] have shown to yield under or over-dosage especially with the most recent delivery techniques [8], [9], [10].

Relying on X-ray imaging to detect and quantify such changes suffers from several limitations. Firstly, as the location and direction of breast swelling may vary, detecting surface deformation with tangential 2D imaging can be challenging and is affected by the gantry angle [8]. Meanwhile, and although Cone Beam Computed Tomography (CBCT) imaging proved more efficient than 2D imaging, the image size is limited and does not suffice in the simultaneous accurate setup of the supraclavicular region and the breast/chest wall [8]. Daily CBCT imaging also involves non-negligible additional dose (in the range of 1 cGy/fraction) which needs to be maintained to the lowest possible level [11], [12]. Lastly, the accuracy of swelling detection and quantification with X-ray based Image-Guided Radiation Therapy (IGRT) is affected by setup uncertainties inherent to conventional tattoo- and laser-based setup.

The present work investigates the use of AlignRT (Vision RT Ltd., UK), a widely used Surface Guided Radiation Therapy (SGRT) solution, as a radiation-free alternative approach to detect and quantify surface changes. SGRT can be used not only for patient positioning, but also to monitor intrafraction motion in free-breathing (FB) or Deep Inspiration Breath Hold (DIBH) treatments. SGRT-guided DIBH has proved to involve a 3 mm isocenter accuracy with respect to IGRT matching [13]. However, while surface changes such as breast swelling are expected to affect the accuracy of SGRT-based setup [13], literature data still lacks a careful analysis of the potential of SGRT in the early detection of anatomy changes.

The primary objective of this work is to benchmark AlignRT’s surface deformation module and determine its accuracy in the detection of morphology changes. A secondary objective involves the development of a clinical flowchart to assist the clinical staff in their decision of repeat simulation and dosimetry planning, using SGRT.

## Materials and Methods

### AlignRT surface deformation module

**Fig. 1:**
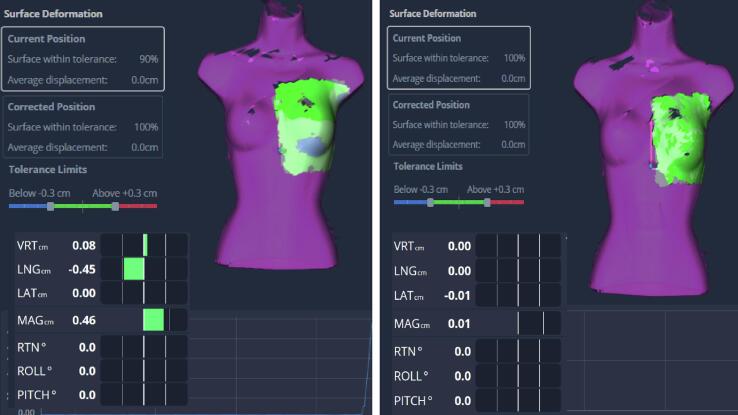
Overview of the surface deformation module in AlignRT Advance v. 6.3 showing the colour map at the current position with embedded RTDs (left) and corrected position (right).

AlignRT is a three-dimensional SGRT system which uses active stereovision to monitor patient motion with sub-mm accuracy [13], [14], [15], [16]. Using an iterative closest point match algorithm, the live surface is registered to the reference surface, and real time deltas (RTDs), representing patient displacement in 6 degrees of freedom, are computed. AlignRT surface deformation module can be used to quantify the extent and amplitude of surface changes (cf. Fig. 1). The module includes a visual representation of the reference surface (purple colour) and the live surface (green colour) inside the Region of Interest (ROI). The computed parameters are average displacement and surface within tolerance inside the ROI. Average displacement (in cm) is the average distance between the ROI of the measured surface and the reference surface. Surface within tolerance (in %) is the percentage of points in the ROI that have a displacement, with respect to the reference surface, inferior to the tolerance limit value. This tolerance limit can be manually adjusted. The colour map is green when both surfaces are within the set tolerance limit, red or blue colour when the live surface is respectively above or below the tolerance limit. Fig. 1 shows surface deformation with the default tolerance limit at ± 0.3 cm for the current and corrected patient position, i.e. once the RTDs are zeroed out.

### Static phantom tests

To validate the surface deformation module, a series of tests were performed using a female torso phantom (The Competitive Store, Santa Ana, USA). This phantom includes fully featured front half form with following dimensions: height 58.4 cm, chest width 25.8 cm, depth 14 cm, and a breast size of 34C. A BOLUSIL® (Kerjean Biotechnologies, Aubergenville, France) water-equivalent bolus of different size and thickness was used to model the oedema. The BOLUSIL® was painted in matte white to avoid any light reflection from shiny surfaces which could negatively impact the optical system.

**Fig. 2:**
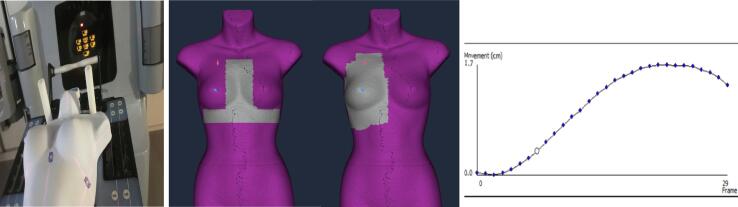
Female torso used for the experiments (left), Postural and Clinical ROI (middle), and dynamic surface captures with regular sampling along the breathing curve (right).

**Fig. 3:**
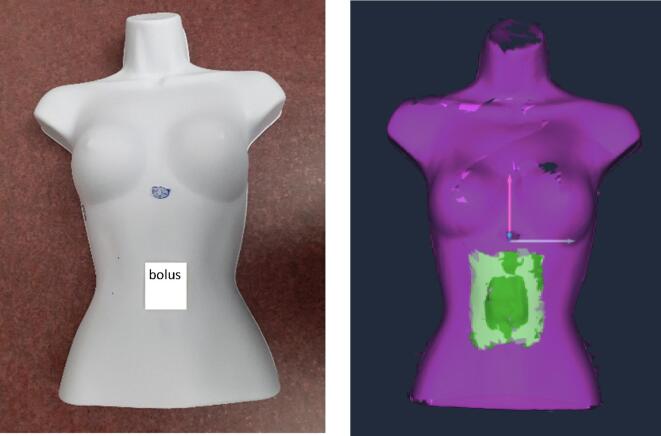
Female torso with the bolus (left), Clinical ROI surrounding the bolus area.

Two ROIs are considered (see Fig. 2). The Postural ROI is used for patient setup as it includes bony areas that deform less than soft tissue. The Clinical ROI (surrounding the entire breast) is used to improve breast positioning but also to detect breast oedema. In this study, the accuracy of oedema detection as a function of bolus thickness and Clinical ROI size is studied. Firstly, a reference surface of the torso was acquired in the absence of any bolus. Next, a 5x5cm^2^ bolus of different thickness (0.5, 1, and 1.5 cm) was set on the torso to simulate swelling (see Fig. 3). The surface deformation module was used to compare the surface captures with/without the bolus. A ROI fitted to the bolus size (5x5cm^2^) was used while three tolerance limits were considered: equal to the bolus thickness, +0.1 cm, and −0.1 cm. Additionally, to investigate the impact of ROI size, a 1 cm-thick bolus with an area of 5x5cm^2^ was placed on the fixed torso phantom while six ROI sizes were considered: 1) 2.5x2.5 cm^2^, 2) 5x5cm^2^, 3) 5x10cm^2^, 4) 10x10cm^2^, 5) 5x20cm^2^, and 6) a clinical ROI of ∼ 15x15cm^2^.

In Fig. 3 shows the bolus on the torso phantom and Clinical ROI around the bolus area.

### Dynamic phantom tests

To determine the impact of patient breathing on the surface deformation module, the torso phantom is fixed on an in-house moving engine to mimic a patient’s breathing pattern with an amplitude of ∼ 1.7 cm and a period of 8 s. A 5x5cm^2^ bolus of 0.5, 1, and 1.5 cm thickness was used with a ROI overlaying the bolus area. Next, dynamic surface captures were acquired across the entire breathing cycle (cf. Fig. 2 right). The reference surface was considered to be that of end exhale. The live surface with the bolus was then successively acquired at six different positions within the breathing cycle corresponding to amplitudes of 0, 0.17, 0.34, 0.5, 0.67 and 0.84 cm. Surface deformation was hence analysed for each of these positions with respect to the end exhale reference position.

## Results

### Static phantom validation

Table 1 summarizes the results of the surface deformation analysis for three different bolus thicknesses. It shows an average displacement value underestimating bolus thickness by 10–17% regardless of the set tolerance limit. Iteratively changing the tolerance limits and analysing the surface percentage within tolerance provide a better estimate of swelling thickness. Namely, for the 0.5 cm-thick bolus, the surface percentage within tolerance increases from 43% to 99% for a tolerance limit of 0.5 or 0.6 cm respectively, indicating that the bolus thickness is between these two tolerance limit values. The same observation holds for the 1 cm-thick bolus (tolerance value between 1 and 1.1 cm) while this was not the case for the 1.5 cm-thick bolus where the surface within tolerance remained well below 90% even with a tolerance limit set to 1.6 cm.

**Table 1:** Average displacement value (cm), tolerance limit (cm) and surface within tolerance (%) for the three bolus thicknesses considering a ROI fitted to the bolus size (5x5cm^2^).

Bolus thickness (cm)	Average displacement (cm)	Relative error (%)	Tolerance limit (cm)	Surface within tolerance (%)	Tolerance limit (cm)	Surface within tolerance (%)	Tolerance limit (cm)	Surface within tolerance (%)
0.5	0.45	−10	±0.4	35	±0.5	43	±0.6	99
1	0.83	−17	±0.9	48	±1	50	±1.1	95
1.5	1.24	−17	±1.4	52	±1.5	54	±1.6	74

**Fig. 4:**
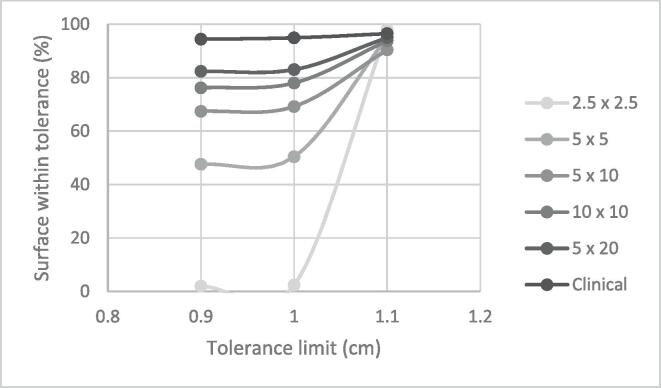
Variation of the percentage of surface within tolerance (%) as a function of tolerance limits (cm) considering a 1 cm-thick, 5x5cm^2^ bolus and different ROI sizes.

Fig. 4 shows the variation of the surface within tolerance as a function of tolerance limit for different ROI sizes considering a 1 cm-thick bolus of 5x5cm^2^. Indeed, with a ROI (2.5x2.5 cm^2^) smaller than the bolus area (5x5cm^2^), the surface percentage within tolerance increased from 2.5% with a 1 cm tolerance limit to 98% with a 1.1 cm tolerance limit. In opposition, the surface percentage within tolerance (at all tolerance limit values) for the clinical ROI remained within 94%.

**Fig. 5:**
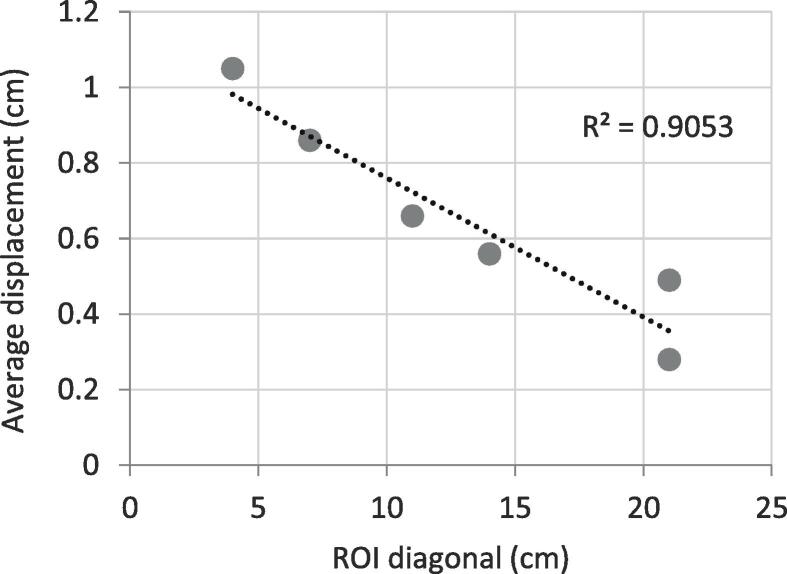
Average displacement (cm) as a function of ROI diagonal for a bolus thickness of 1 cm and bolus size of 5x5cm^2^.

Fig. 5 shows that the average displacement increases linearly with decreasing ROI diagonal with a fitting of R^2^ > 0.9. For a ROI (2.5x2.5 cm^2^) smaller than the bolus size (5x5cm^2^), the average displacement (1.05 cm) provides an accurate estimate of the oedema thickness (within 5%). In opposition, with a clinical ROI, the 1 cm-thick bolus was largely underestimated with an average displacement value of 0.28 cm (-72%).

### Dynamic phantom validation

**Fig. 6:**
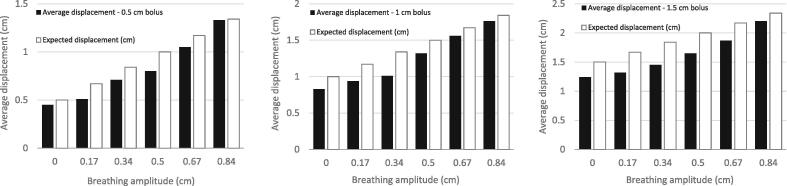
Comparison of computed average displacement (cm) and expected displacement (cm) as a function of phantoms’ breathing amplitude (cm) for the 0.5 cm (left), 1 cm (middle) and 1.5 cm-thick bolus (right).

**Fig. 7:**
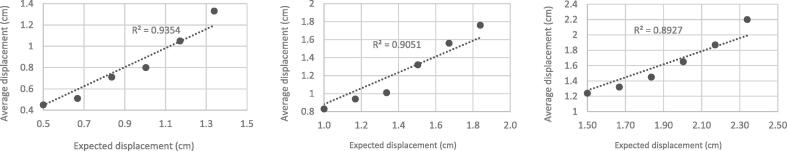
Computed average displacement (cm) as a function of expected displacement (cm) for the 0.5 cm (left), 1 cm (middle) and 1.5 cm-thick bolus (right).

Table 2 shows the impact of patient breathing on the average displacement value and on the surface discrepancy percentage. It clearly demonstrates that the comparison of the reference surface to the live surface should occur at the same breathing amplitude/phase otherwise the swelling estimate could be misleading. When the chest elevation with breathing is taken into account, Table 2 as well as Fig. 6 show that the average displacement computed by the surface deformation module and the expected swelling thickness (bolus thickness + chest elevation due to breathing) fit within 24% with the average displacement systematically underestimating the swelling. Similar results are observed for all three bolus thicknesses (cf. Fig. 6). When plotting the expected vs. computed swelling thickness, a linear fitting function associated with a correlation coefficient R^2^ > 0.99 was obtained for all three bolus thicknesses which further validates the approach based on dynamic captures to efficiently use the surface deformation module (cf. Fig. 7).

**Table 2:** Average displacement (Avg. displ.) value (cm), tolerance limit (cm) and surface within tolerance (%) as a function of breathing amplitude and bolus thickness considering a ROI fitted to the 5x5cm^2^ bolus area.

Breathing amplitude (cm)	Bolus thickness 0.5 cm			Bolus thickness 1 cm			Bolus thickness 1.5 cm		
	Avg. displ. (cm)	Tolerance limit (cm)	Surface within tolerance (%)	Avg. displ. (cm)	Tolerance limit (cm)	Surface within tolerance (%)	Avg. displ. (cm)	Tolerance limit (cm)	Surface within tolerance (%)
0	0.45	±0.6	99	0.83	±1.1	95	1.24	±1.6	74
0.17	0.51		84	0.94		84	1.32		52
0.34	0.71		28	1.01		42	1.45		48
0.5	0.8		25	1.32		37	1.65		47
0.67	1.05		1.3	1.56		27	1.87		42
0.84	1.33		0.8	1.76		20	2.20		31

### Clinical implementation

**Fig. 8:**
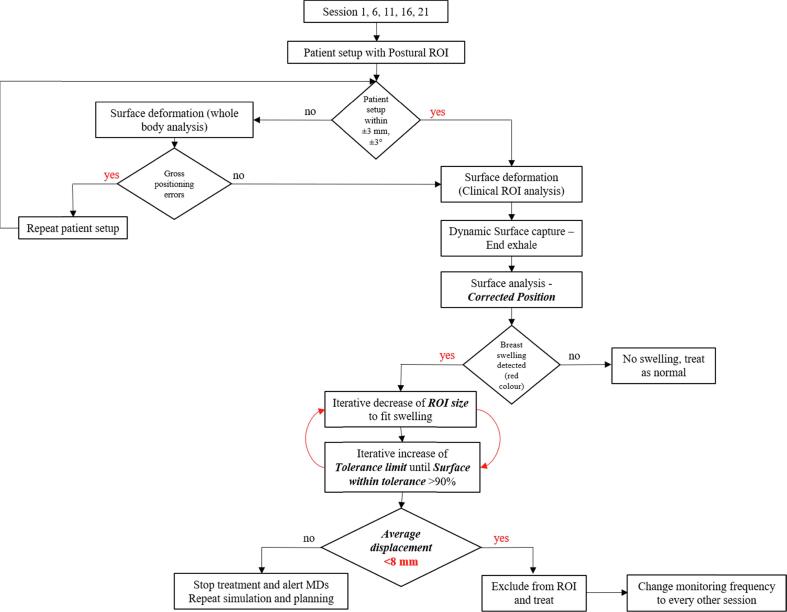
Flowchart description of the oedema detection strategy using the surface deformation module.

Based on the present study, the flowchart described in Fig. 8 was proposed to detect and quantify breast swelling from the early stages prior to X-ray imaging. The procedure shall be applied on weekly basis in the absence of any correlation between swelling occurrence and time from the first fraction [7]. In line with local dosimetry margins for breast irradiations, if the oedema thickness exceeds 8 mm, patient treatment is stopped, physicians are alerted and shall decide on repeat simulation and dosimetry planning. If the swelling does not exceed the 8 mm dosimetry margin, the ROI is adjusted to exclude the swelling and the monitoring frequency is increased from weekly basis to every other treatment session.

## Discussion

When benchmarking the surface deformation module with three bolus of different thicknesses (0.5, 1, 1.5 cm), it was found that ROI size greatly affects the performance of the system with a negative linear correlation between average displacement and ROI diagonal (R^2^ > 0.92). ROI should hence be iteratively reduced to fit the swelling area for accurate estimate of its thickness. Additionally, while the surface percentage within tolerance was at 98% and 95% for the 0.5 cm and 1 cm-thick bolus respectively, this value was as low as 74% for the 1.5 cm-thick-bolus even with a tolerance limit set to 1.6 cm. This is likely due to the impact of such a thick bolus on the RTD values, which exceed the default 0.3 cm motion threshold set in AlignRT. Such a result indicates that large breast swellings are likely to prevent accurate patient positioning, alerting hence the users to possible anatomical changes even with clinical ROIs largely exceeding the size of the swelling. In this case, it is critical to assess average displacement after the RTD values are zeroed out, i.e. in the corrected position window (cf. Fig. 1 and Appendix A).

The impact of patient breathing on average displacement values showed a linear correlation between average displacement values and vertical shifts (cf. Fig. 7). Taking dynamic surface captures proved efficient in minimizing such uncertainties with a systematic analysis at end exhale. However, changes of the patient baseline position (i.e. end exhale) which may occur in routine clinical conditions are likely to induce minimal impact on the correspondence between computed and expected displacement (cf. Fig. 6). Extrapolation of the obtained results to higher breathing amplitudes (eg. DIBH), should be confirmed with further measurements.

Based on this work, several improvements to the surface deformation module can be suggested. Firstly, in addition to the average displacement value within the ROI, computing the maximum displacement value and plotting the topographic profile of the swelling would be beneficial. Secondly, giving the user the flexibility to run the surface deformation module on a specific area of the surface/patient instead of having to change the ROI iteratively to fit to the target/swelling area would be helpful. Thirdly, automating the iterative adjustment of ROI size and tolerance limits to the swelling area would help avoiding time consuming steps and eliminate any user-induced bias with an analysis at the Mesh level. Fourthly, the extent and size of the surface deformation/swelling is not quantified and should be given in cm^2^. A ruler would allow the user to measure the ROI size, swelling dimensions, etc. and determine its position/distance with respect to relevant anatomical landmarks/structures. Lastly, offering the user more features to track the evolution of oedema over time would be invaluable.

The present study also involves some limitations. Firstly, the static phantom represents one unique breast topography and does not replicate different breast shapes, sizes and morphologies. The phantom also does not enable to reproduce a chest wall treatment where the resolution of post-operative seroma and tissue shrinking may also affect treatment delivery. Nonetheless, such a phantom and topography is sufficient to benchmark and validate the surface deformation module for detecting and quantifying breast swelling. Secondly, the dynamic phantom tests did not include variable breathing amplitudes to investigate the accuracy of the surface deformation tool. Nonetheless, the conservative approach introduced here to manage patient breathing based on dynamic surface captures should allow for good estimate of tissue swelling at end exhale phase. Finally, the clinical implementation of the node detection process/flowchart can be optimized to adjust the analysis frequency from a weekly basis to a patient-specific and individualized approach. Additionally, the threshold value (i.e. 8 mm) used to trigger patient re-simulation and planning solely relies on oedema thickness (i.e. average displacement detected with AlignRT) and local clinical and dosimetry margins. More work is needed to take into consideration the size/extent of a real patient oedema and its location on the breast as well as the delivery technique (Conformal vs. IMRT vs. VMAT, FB vs. DIBH).

## Conclusion

This phantom study benchmarked AlignRT’s surface deformation module to detect and quantify the occurrence of oedema with sub-mm precision. Using SGRT, a clinical flowchart allowing for a radiation-free weekly monitoring of any surface changes was proposed to help physicians and physicists in their decision of repeating patient simulation and planning. Several software improvements were highlighted to further ease the use of such a tool.

Future work will focus on testing this deformation module on real patients and comparing the results to CBCT data.

## Declaration of Competing Interest

The authors declare that they have no known competing financial interests or personal relationships that could have appeared to influence the work reported in this paper.

## Figures and Tables

**Table A1:** Average displacement value (cm), tolerance limit (cm) and surface within tolerance (%) as a function of patient positioning shifts considering a ROI fitted to the 0.5 cm-thick 5x5cm^2^ bolus area.

Positioning shifts		Avg displacement (cm)	Tolerance limit (cm)	Surface within tolerance (%)	Tolerance limit (cm)	Surface within tolerance (%)	Tolerance limit (cm)	Surface within tolerance (%)
Vertical (cm)	0.1	0.67	±0.4	10	±0.5	14	±0.6	19
	0.2	0.73		12		16		21
	0.3	0.81		9		12		16
Longitudinal (cm)	0.1	0.53		10		17		54
	0.2	0.52		11		17		53
	0.3	0.52		10		16		53
Lateral (cm)	0.1	0.57		11		17		51
	0.2	0.58		9		15		52
	0.3	0.57		11		16		51
Yaw (°)	0.1	0.49		28		33		96
	0.2	0.48		29		33		94
	0.3	0.49		28		32		94

## Appendix A - uncertainty burden from patient misalignment

To study the impact of inevitable patient misalignment on the surface deformation module, gradual displacement of the torso phantom was operated in all three translational directions (1, 2, and 3 mm in line with the patient setup tolerances considered in local routine conditions) and in yaw rotation (1, 2 and 3° also in line with local patient setup tolerances).

Results show that only shifts in the vertical direction affect the average displacement calculation (cf. Table A1). The latter remained unaffected even when lateral shifts of 3 mm or yaw rotations of 3° were implemented. Meanwhile, when compared to data shown in Table 1, surface percentage within tolerance proved to be greatly affected by any displacement possibly yielding gross errors in quantifying the extent and thickness of the swelling if not accounted for. Similar results were obtained with the 1 cm and 1.5 cm-thick boluses. Nonetheless, the surface deformation module computes average displacement and surface percentage within tolerance both for the current patient position and once patient misalignment are corrected for i.e. once the real time deltas shifts are applied (cf. Fig. 1). As such, no additional uncertainties to the swelling detection and quantification can be attributed to the original patient misalignment.
